# Personal

**Published:** 1897-04

**Authors:** 


### Personal.

Dr. J. Chalmers DaCosta, of Philadelphia, was recently elected clinical professor of surgery at Jefferson Medical College. Dr. DaCosta has been connected with the college for many years and has until lately been demonstrator of surgery and chief of the out-patients' department. The new appointment is made in recognition of his long service and valuable contributions to surgical literature.

---

Dr. Edward Clark, of Buffalo, whose practice is limited to diseases of the rectum, has removed his office from 180 West Chippewa street to 854 Ellicott Square. Hours: 1–3 p. m. Sundays, by appointment. Telephone, Seneca 1154. His residence is 2268 Main street.

---

Dr. George F. Cott, of Buffalo, whose practice is limited to diseases of the nose and throat, has removed his office to the Mooney-Brisbane building, corner of Main and Clinton streets, room 531, fifth floor. Hours: 10 a. m.–2 p. m. Telephone, Seneca 1201.

---

Dr. Horace Clark, of Buffalo, whose practice is limited to diseases of the nose, throat and ear, has removed his office to 846 Ellicott Square. Hours: 9 a. m.–4 p. m. Telephone, Seneca 92. His residence is No. 21 West North street. Telephone, Bryant 125.

---

Dr. Frank J. Thornbury, of Buffalo, whose practice is limited to diseases of the skin, announces his removal from 405 Delaware avenue to The Colonial, 401 Delaware avenue. Hours, 1, and 4 to 6 p. m. Telephone, The Colonial.

Dr. William C. Krauss, of 382 Virginia street, Buffalo, has removed to 371 Delaware avenue. Office hours, 8 to 10 a. m., 2 to 4, and 7 to 8 p. m. Telephone, Tupper 101.

---

Dr. A. B. Wilson, of Buffalo, announces his removal from 345 Virginia street to 78 Fargo avenue. General practice. Telephone, Tupper 425.

